# Analysis of adverse events related to extracorporeal membrane oxygenation from a nationwide database of patient-safety accidents in Japan

**DOI:** 10.1007/s10047-023-01386-z

**Published:** 2023-02-16

**Authors:** Hiroki Hadano, Tadashi Kamio, Kiyomitsu Fukaguchi, Mizuki Sato, Yumiko Tsunano, Hiroshi Koyama

**Affiliations:** Division of Critical Care, Shonan Kamakura General Hospital, 1370-1, Okamoto, Kamakura-shi, Kanagawa 247-8533 Japan

**Keywords:** Extracorporeal membrane oxygenation, Adverse events, Complications, Patient safety, Risk management

## Abstract

Although adverse events related to extracorporeal membrane oxygenation have been reported, epidemiological data on life-threatening events are insufficient to study the causes of such adverse events. Data from the Japan Council for Quality Health Care database were retrospectively analyzed. The adverse events extracted from this national database included events associated with extracorporeal membrane oxygenation reported between January 2010 and December 2021. We identified 178 adverse events related to extracorporeal membrane oxygenation. At least 41 (23%) and 47 (26%) accidents resulted in death and residual disability, respectively. The most common adverse events were cannula malposition (28%), decannulation (19%), and bleeding (15%). Among patients with cannula malposition, 38% did not undergo fluoroscopy-guided or ultrasound-guided cannulation, 54% required surgical treatment, and 18% required trans-arterial embolization. In this epidemiological study in Japan, 23% of the adverse events related to extracorporeal membrane oxygenation had fatal outcomes. Our findings suggest that a training system for cannulation techniques may be needed, and hospitals offering extracorporeal membrane oxygenation should perform emergency surgeries.

## Introduction

Extracorporeal membrane oxygenation (ECMO) supports patients with cardiac or respiratory failure in an intensive care unit. According to the Extracorporeal Life Support Organization (an international voluntary registry) (ELSO), the use of ECMO has increased over the past decade [1]. The coronavirus disease 2019 (COVID-19) pandemic has significantly increased the number of patients with severe respiratory failure requiring ECMO [2]. The ELSO guidelines provide information on cannulation during ECMO and managing patients undergoing ECMO [2–4]. Although ECMO should be initiated and maintained safely, life-threatening adverse events related to ECMO have been widely reported [5–14].

Bleeding was the most frequently reported adverse event in two systematic reviews on veno-arterial and veno-venous ECMO [5, 6]. However, the reviews revealed that almost all adverse events were reported at high-volume centers; therefore, sampling bias cannot be excluded. According to the Japan Society of Extra-Corporeal Technology in Medicine, adverse events related to ECMO occurred in 4.0% of the patients, with severe adverse events occurring in 0.44% (https://jasect.org/1463). However, these data were collected using online questionnaires, which were designed to record only the number of accidents. Therefore, to avoid sampling bias and to clarify how these accidents occur, we used an accident-reporting system to collect data for this study. To the best of our knowledge, no study has been conducted on the causes of ECMO-related adverse events using data from an accident-reporting system.

According to the World Health Organization Draft Guidelines for Adverse Event Reporting and Learning Systems, the most important goal of a reporting system is to understand the causes and consequences of accidents; these form the cornerstone for patient-safety improvement [15]. Since 2004, the Japan Council for Quality Health Care Division of Adverse Event Prevention has been collecting information on medical near-misses and adverse events to improve the quality of healthcare services (https://www.med-safe.jp/contents/english/index.html).

Therefore, we analyzed data from a nationwide database of patient-safety accidents related to ECMO. Our primary aim was to compile epidemiological data on ECMO-related accidents. Our secondary aim was to analyze the risk factors for life-threatening ECMO-related adverse events.

## Materials and methods

### Study design and settings

This study employed a descriptive qualitative approach. Data were collected from patient-safety accident reports recorded in a Japanese database between January 2010 and December 2021.

### Features of the database used

Since 2004, the Japan Council for Quality Health Care has been conducting various activities to maintain public confidence in healthcare services as well as improve the quality of these services. These activities include evaluating medical services and the Project to Collect Medical Near-Miss/Adverse Event Information. As of December 31, 2021, 1,575 medical institutions (approximately 20% of the total number of hospitals in Japan) were registered with this project. Although participation in this project is mandatory for tertiary teaching hospitals (such as medical universities, national hospitals, and hospitals providing advanced treatments), other medical institutions have joined the project voluntarily. Overall, 273 hospitals (138,150 inpatient beds) were required to participate in this project from December 31, 2021. In total, 47,527 cases of medical adverse events were reported between October 2004 and December 2021, and 4,674 have been reported between January and December 2021. Information regarding medical adverse events is published quarterly and annually. Additionally, annual reports have been available to interested parties since 2010 on the project’s website (http://www.med-safe.jp/contents/english/index.html).

The following medical adverse events must be reported: (a) apparent errors in treatment or management that resulted in the patient’s mental/physical disability or death or those that required unexpected treatment, treatment to an unexpected extent, or other medical procedures; (b) unapparent errors in treatment or management that resulted in the patient’s mental/physical disability or death or those that required unexpected treatment, treatment to an unexpected extent, or other medical procedures (including events possibly associated with the treatment or management provided, limited to unexpected events); and (c) errors other than those described in (a) and (b) or any information conducive to preventing medical adverse events and their recurrence at medical institutions. Bleeding complications involving the brain and gastrointestinal tract may be relatively less reported in this system. Therefore, our study primarily evaluated adverse events related to “errors” in the ECMO procedures.

We used anonymized data from the database that were unlinked to individual patient information. The need for ethical approval and informed consent was waived because of the study’s retrospective nature and the anonymity of the analyzed data.

### Database search methods

We searched the database for all ECMO-associated adverse events that were reported between January 2010 and December 2021; a free-text search was conducted to identify procedure-specific events. Because this database is only available in Japanese, the Japanese words for “ECMO,” “artificial cardiopulmonary device,” and “percutaneous cardiopulmonary support” were used for data extraction. In the event of a duplicate report, the data were integrated.

We collected the following data from the database: patient sex and age, location, clinical experience of the primary operator (in years), adverse events, causes of adverse events, and the reporter’s assessment of the possibility of residual disability. Based on the level of harm anticipated by the reporter, the accidents were then classified as follows: (a) event resulting in death, (b) event with a high potential for residual disability, (c) event with a low potential for residual disability, (d) event without a potential for residual disability, and (e) unknown. Two authors (HH and TK) independently reviewed the accident reports to determine their eligibility for inclusion and classified them by reviewing their free-text descriptions. Because of our focus on ECMO-associated adverse events, we excluded adverse events unrelated to ECMO and those related to mechanical cardiopulmonary support only during surgery.

We categorized ECMO-related adverse events into cannula malposition, accidental decannulation, bleeding, air in the circuit, thromboembolism, limb ischemia, gas supply issue, foreign body remnants, and others. Cannula malposition was defined as extravascular cannulation or cannulation of the wrong vessel (such as arterial-arterial and venous-venous cannulation). In contrast, decannulation was defined as accidental cannula removal that required reinsertion or position adjustment. Bleeding was defined as bleeding during or after managing patients undergoing ECMO. Therefore, bleeding due to extravascular cannulation was classified as cannula malposition, whereas bleeding or air in the circuit due to accidental decannulation was classified as decannulation.

### Outcome measures

First, we examined which adverse events were more likely to lead to death or had a high potential for residual disability. Second, we focused on cannula malposition and analyzed the patient’s age, types of ECMO, the vessel of cannulation, site of injury, physician’s department, physician’s experience (in years), location, and whether the cannulation was fluoroscopy- or ultrasound-guided. We differentiated extracorporeal cardiopulmonary resuscitation from the veno-arterial-ECMO since the cannulation techniques may differ. The ELSO guideline recommends a cut-down technique for cannula insertion as the first choice in patients with extracorporeal cardiopulmonary resuscitation because of no detection of femoral artery pulse [4]. In contrast, the percutaneous cannulation technique is recommended in veno-arterial-ECMO. Third, we focused on accidental decannulation and analyzed the level of harm, the type of cannula, the cause, and the considered factors. Fourth, we analyzed the bleeding site, causes of air in the circuit, and additional treatments administered for bleeding, the air in the circuit, and thromboembolism.

## Results

**Fig. 1:**
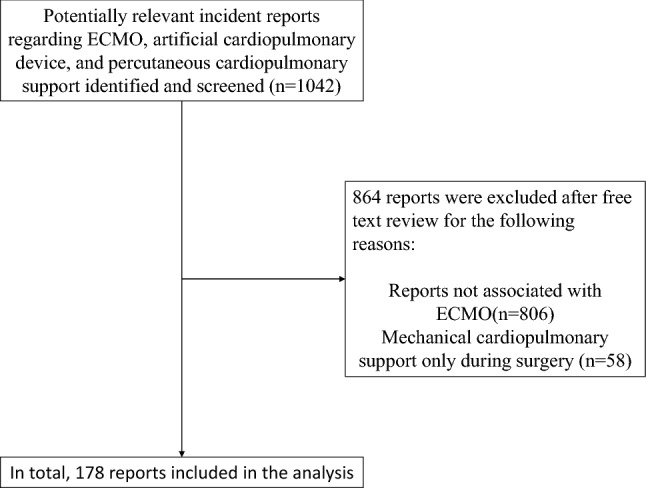
Flow diagram showing the selection of accident reports. Details are shown regarding the database search and the number of reports screened. Reports unrelated to extracorporeal membrane oxygenation and those that did not meet our inclusion criteria were excluded. *ECMO* extracorporeal membrane oxygenation

We identified 1,042 cases of adverse events that were potentially relevant to this study; those involving unrelated-ECMO events or related to mechanical cardiopulmonary support only during surgery were excluded. Therefore, 178 cases with ECMO-related adverse events were included in our analyses (Fig. 1).

**Fig. 2:**
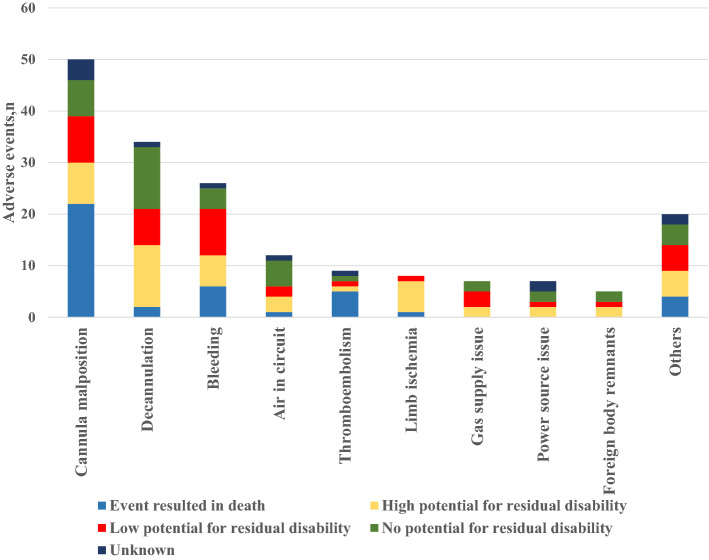
Characteristics and level of harm of the adverse events

Figure 2 shows the flow diagram of the report analysis. The most common ECMO-related adverse events were cannula malposition (28% [50/178]), followed by decannulation (19% [34/178]), bleeding (15% [26/178]), air in the circuit (7% [12/178]), thromboembolism (5% [9/178]), limb ischemia (4% [8/178]), gas supply issue (4% [7/178]), power supply issue (4% [7/178]), and foreign body remnants (3% [5/178]). Furthermore, when classified using the level of harm, 41 (23%), 47 (26%), 78 (44%), and 12 (7%) adverse events were categorized as those resulting in death, with a high potential for residual disability, with a low potential for residual disability, and unknown, respectively. Moreover, 44% [22/50] and 56% [5/9] of cannula malposition and thromboembolism events were classified as resulting in death, respectively. Finally, 54% (97/178) and 33% (58/178) of the patients were ˃ 60 and ˃ 70 years, respectively.

Table 1 summarizes the adverse events according to cannula malposition; 74% (37/50) of the patients with cannulation malposition were aged ˃ 60 years. The ECMO types were extracorporeal cardiopulmonary resuscitation, veno-arterial-ECMO, and veno-venous-ECMO in 46% (23/50), 28% (14/50), and 26% (13/50) of these patients, respectively. However, cases of central ECMO were absent, because we excluded patients who received mechanical cardiopulmonary support only during surgery. The most common insertion site causing cannula malposition was the right internal jugular vein (20% [10/50]). The retroperitoneum or the intraperitoneal structures were the most common injury sites (63% [27/50]). Here, the cannula was inserted via the femoral blood vessels in all patients with injuries. Conversely, the mediastinal or thoracic structures were the most common injury sites when the cannula was inserted via the right internal jugular vein. Furthermore, 38% (19/50) of the patients had neither fluoroscopic nor ultrasound guidance at ECMO induction. Among the patients with cannula malposition, 54% (27/50) required surgical treatment, 18% (9/50) required trans-arterial embolization, and 26% (13/50) died before treatment.

**Table 1:** Summary of adverse events resulting from cannula malposition (total number of cases = 50)

Characteristic	Number, *n* (%)
Patient age, years	
0–9	1 (2%)
10–19	2 (4%)
20–59	10 (20%)
60–69	14 (28%)
70–79	15 (30%)
≥ 80	8 (16%)
Types of ECMO	
Extracorporeal cardiopulmonary resuscitation	23 (46%)
Venoarterial-ECMO	14 (28%)
Veno-venous-ECMO	13 (26%)
Vessel of insertion	
Right femoral vein	9 (18%)
Left femoral vein	9 (18%)
Right femoral artery	4 (8%)
Left femoral artery	7 (14%)
Right internal jugular vein	10 (20%)
Unknown femoral blood vessel	4 (8%)
Wrong vessel	7 (14%)
Injury site	
Retroperitoneal or intraperitoneal structures	27 (54%)
Mediastinal or thoracic structures	9 (18%)
Cannulation site	4 (8%)
Cardiac injury	3 (6%)
Wrong vessel	7 (14%)
Injury site from the femoral vein or artery	
Retroperitoneal or intraperitoneal structures	27 (82%)
Cannulation site	4 (12%)
Mediastinal or thoracic structures	2 (6%)
Injury site from the right internal jugular vein	
Mediastinal or thoracic structures	8 (80%)
Cardiac site	2 (20%)
Operator specialty	
Emergency intensive care	21 (36%)
Cardiology	20 (34%)
Cardiovascular surgery	15 (26%)
Thoracic surgery	2 (3%)
Operator’s clinical experience, years	
1–5	7 (14%)
6–10	17 (34%)
11–15	11 (22%)
16–20	4 (8%)
≧ 21	11 (22%)
Care setting	
Emergency room	18 (36%)
Catheter laboratory	12 (24%)
Intensive care unit	12 (24%)
Operating room	6 (12%)
General care ward	2 (4%)
Guidance	
None	19 (38%)
Fluoroscopic	16 (32%)
Ultrasound	7 (14%)
Ultrasound and fluoroscopic	0 (0%)
Unknown	8 (16%)
Additional treatment	
Surgical repair	27 (54%)
Trans-arterial embolization	9 (18%)
Death before treatment	13 (26%)

*ECMO* extracorporeal membrane oxygenation

Table 2 summarizes the adverse events arising from decannulation. All patients experienced complete cannula removal and required reinsertion. The arterial cannula was removed in 85% (29/34) of the patients. In 41% (14/34) of the patients, removal occurred while the physician performed another procedure (such as surgery and percutaneous coronary intervention). Insufficient fixing (44% [23/34]), blinded inserting positions (41% [14/34]), and inadequate communication (21% [7/34]) were the possible causes of cannula removal.

**Table 2:** Characteristics of cases of decannulation (total number of cases = 34)

Characteristic	Number, *n* (%)
Level of harm	
Complete decannulation	34 (100%)
Incomplete decannulation	0 (0%)
Type of cannula	
Arterial	29 (85%)
Venous	3 (9%)
Additional arterial cannula of the lower extremity	2 (6%)
Cause	
Physician performed another procedure	14 (41%)
Position change	11 (32%)
Transportation	4 (12%)
Body motion	3 (9%)
Unknown	2 (6%)
Considered factor	
Insufficient fixing	23 (44%)
Blinded inserting position	14 (27%)
Lack of communication	7 (13%)
Insufficient sedation	5 (10%)
Obese patient	3 (6%)

Table 3 shows the characteristics of bleeding during or after ECMO, the air in the circuit, and thromboembolism. Of the 26 patients with bleeding, 42% (11/26) and 35% (9/26) had bleeding from another procedure site and due to circuit damage (for example, a fissure in the connection between the arterial cannula and the circuit and cracks in flow sensor), respectively. Regarding the duration of circuit damage, two cases occurred on the first day of ECMO, one on the second day, and another six were unknown when they occurred. Incorrect clamp release (33% [4/12]), incorrect three-way stopcock operation (33% [4/12]), and machine breakage (17% [2/12]) were considered the causes of air in the circuit. For the duration of machine breakage, one case occurred on the first day of ECMO, and one was unknown when it occurred. Bleeding in 38% (11/26) of the patients required surgical repair. Emergency circuit change was required in 15% (4/26), 50% (6/12), and 67% (6/9) of the patients with bleeding, the air in the circuit, and thromboembolism, respectively.

**Table 3:** Characteristics of bleeding during or after ECMO, the air in the circuit, and thromboembolism

Characteristic	Number, *n* (%)
Site of bleeding during or after ECMO (total number of cases = 26)	
Another procedure site	11 (42%)
Circuit damage	9 (35%)
Site of removal	3 (12%)
Additional treatment for bleeding during or after ECMO	
Surgical repair	10 (38%)
Circuit change	4 (15%)
Death before treatment	3 (12%)
Cause of air in circuit (total number of cases = 12)	
Incorrect clamp release	4 (33%)
Incorrect three-way stopcock operation	4 (33%)
Machine breakage	2 (17%)
Additional treatment for air in the circuit	
Circuit change	6 (50%)
Air removal	6 (50%)
Additional treatment for thromboembolism (total number of cases = 9)	
Circuit change	6 (67%)
Death before treatment	3 (33%)

*ECMO* extracorporeal membrane oxygenation

## Discussion

Here, we identified 178 ECMO-related accidents between 2010 and 2021 in the Japan Council for Quality Health Care’s open database. The most common adverse events were cannula malposition, decannulation, bleeding, the air in the circuit, and thromboembolism. Of all accidents, 49% caused severe harm (i.e., residual disability or death).

A previous study reported that the overall pooled estimate of vascular adverse events related to ECMO was 16.7–29.5% [7, 8, 16]. Although the rate of cannula malposition was unknown in our study, it was the most common adverse event. According to the ELSO guidelines, age < 70 years is an indication for extracorporeal cardiopulmonary resuscitation [4], and older age is a relative contraindication for veno-venous ECMO (no threshold has been established) [3]. However, ECMO is frequently initiated in older individuals in Japan because of its super-aging society [17]. Iatrogenic vascular injuries appear to be more common in older patients with calcified atherosclerotic disease [18], as was observed in this study. According to the ELSO guidelines, no cannulation site is strongly preferred [4]. Our findings did not indicate whether the cannulation site is likely to cause vascular adverse events. However, the number of adverse events may also not depend on the physician’s years of experience. Compared with unguided cannulation, combined ultrasound- and fluoroscopy-guided cannulation was reported to lead to a lower incidence of cannula malposition [19]. Although cannula malposition was performed under fluoroscopic or ultrasound guidance alone in most cases, a combined approach was not used in any case. Both ultrasound and fluoroscopic guidance are recommended during ECMO induction, particularly in older patients who are more prone to cannula malposition. However, some patients experienced cannula malposition despite fluoroscopy confirming that the guidewire was in the correct position. Therefore, training in the cannulation technique may be considered in future training programs at high-volume ECMO centers to prevent and reduce adverse events.

Accidental decannulation is considered a catastrophic adverse event. Arterial and venous decannulation cause massive bleeding and air in the circuit, respectively. Although Kim et al. reported that decannulation occurred in 1.3% of their cases, they could not determine the related causes [9]. Our study showed that decannulation occurred when doctors performed another procedure and when the nurses changed the patient’s position; the nurses’ attention was then distracted from the cannulation site during ECMO initiation. Therefore, assessing the insertion site at such times may reduce the number of ECMO-associated accidents. Moreover, the common reasons for decannulation were insufficient fixing and a blinded inserting position. In Kim et al.’s study, most (90.9%) of the mechanical life-threatening events required circuit and cannula change [9]. However, in our study, 100, 50, and 67% of the patients with decannulation, the air in the circuit, and thromboembolism required a circuit change, respectively. Some hospitals did not have clinical engineers on the night shift, and emergency circuit changes could not be performed. Furthermore, 38% of the patients with bleeding during or after ECMO required surgical repair. Therefore, hospitals, where ECMO can be induced should be limited to those with surgeons and clinical engineers who are available 24 h per day; the number of hospitals where ECMO can be safely performed should be limited to those with suitable facilities.

This study had several strengths. To the best of our knowledge, this is the first investigation into ECMO using data from an accident-reporting system. By extracting data from accident reports, we could gather detailed data on the attending physician’s years of experience and the circumstances of each reported accident. Because ECMO-related adverse events cause severe harm, fear of blame and retaliation and a sense of guilt develop easily, which may deter meaningful reporting [20]. Additionally, the number of patients who undergo ECMO is limited. Therefore, there are few reports of accidents related to this procedure. However, we included 178 ECMO-related accidents. Moreover, the free-text nature of the accident reports enabled us to identify the causes of the accidents. Historically, bleeding is the most feared and frequent adverse event with ECMO [5]. However, data from the accident-reporting system used in this study suggest that cannula malposition and decannulation are more common adverse events, and cannula malposition and thromboembolism are more feared.

This study had some limitations. First, it used a retrospective design and analyzed data from an existing database, implying that the conclusions are necessarily limited in their application and causality cannot be determined. Second, although ˃ 1,500 medical institutions have joined this national health database, the accuracy of the reporting system depends on individual professionals. Moreover, the adverse events reported in this database were mostly related to human errors. Bleeding is a common adverse event with ECMO [5, 6]; however, adverse events may be relatively less reported in this system. Therefore, our results may underestimate the actual occurrence of adverse events. Third, in the United Kingdom, hospital accident reporting is a component of individual hospitals’ risk governance processes [21], and all hospitals are required to report to a national reporting system. In Japan, approximately 20% of all hospitals are registered in the reporting system we used for this study. Nevertheless, we extracted all our data from the database and conducted a free-text search to obtain accurate information. Therefore, we included 178 accidents. Fourth, we could not calculate the incidence rate because it is impossible to obtain the actual dominator. However, it is important to have epidemiological data for life-threatening adverse events associated with ECMO. Fifth, a clear and accurate definition for each adverse event is difficult, leading to some implications. Although two authors (HH and TM) reviewed all reports as we described previously, a subjective bias of categorization might exist. Finally, ECMO equipment is evolving to prevent adverse events while the number of ECMO cases is increasing. Given the 10-year study period, some data used are ˃ 10 years old and may not reflect the current situation.

## Conclusion

ECMO is indicated in acute severe heart or lung failure cases with high mortality risk, and the related adverse events can be fatal. We identified that at least 41 fatal adverse events had occurred, and many cases required emergency surgical repair or circuit change over the 10-year period for which we gathered data. Therefore, our analysis suggests that extreme care should be taken with patients who undergo ECMO to prevent fatal adverse events and be prepared to perform surgery and circuit replacement at any time.

## Data Availability

The data that support the findings of this study are openly available in Japan Council for Quality Health Care at https://www.med-safe.jp/contents/english/index.html.
